# A novel prognostic model to predict prognosis of patients with osteosarcoma based on clinical characteristics and blood biomarkers

**DOI:** 10.7150/jca.105590

**Published:** 2025-03-10

**Authors:** Shulin Chen, Liru Tian, Chuan Li, Dongmei Zhong, Tingting Wang, Yuyu Chen, Taifeng Zhou, Xiaoming Yang, Zhiheng Liao, Caixia Xu

**Affiliations:** 1 Research Center for Translational Medicine, the First Affiliated Hospital, Sun Yat-sen University, 58 Zhongshan Road 2, Guangzhou, Guangdong 510080, P.R. China.; 2 Department of Clinical Laboratory, State Key Laboratory of Oncology in South China, Collaborative Innovation Center for Cancer Medicine, Sun Yat-sen University Cancer Center, Guangzhou 510060, P. R. China.; 3 Department of Pathology, State Key Laboratory of Oncology in South China, Collaborative Innovation Center for Cancer Medicine, Sun Yat-sen University Cancer Center, Guangzhou 510060, P. R. China.; 4 Guangdong Provincial Key Laboratory of Orthopedics and Traumatology, Department of Spine Surgery, the First Affiliated Hospital of Sun Yat-sen University, Guangzhou 510080, China.; Shulin Chen and Liru Tian have contributed equally to this work.

**Keywords:** Osteosarcoma, Blood biomarkers, LASSO-Cox regression, Prognostic

## Abstract

**Purpose:** Osteosarcoma (OSC) is a high-morbidity bone cancer with an unsatisfactory prognosis. Timely and accurate assessment the overall survival (OS) and progression-free survival (PFS) in patients with OSC are required to guide and select the best treatment. This study aimed to develop a simple, convenient and low-cost prognostic model based on clinical characteristics and blood biomarkers for predicting OS and PFS in OSC patients.

**Methods:** Overall, 158 patients with OSC included from the Sun Yat-sen University Cancer Center in this retrospective study. LASSO-Cox algorithm was used to shrink predictive factor size and established a prognostic risk model for predicting OS and PFS in OSC patients. The predictive ability of the survival model was compared to the Tumor Node Metastasis (TNM) stage and clinical treatment by concordance index (C-index), time-dependent receiver operating characteristic (td-ROC) curve, decision curve analysis (DCA), net reclassification improvement index (NRI), and integrated discrimination improvement index (IDI).

**Results:** Based on results from the LASSO-Cox method, gender, family history of cancer, monocyte (M), red blood cell (RBC), lactic dehydrogenase (LDH), and cystatin C (Cys-C) were identified to construct a novel predictive model for the OSC patients. The C-index of the prognostic model to predict OS and PFS were 0.713 (95% CI = 0.630 - 0.795) and 0.636 (95% CI = 0.577 - 0.696), respectively, which were higher than the OS and PFS of TNM stage and clinical treatment. Td-ROC curve and DCA of the predictive model also demonstrated good predictive accuracy and discriminatory power of OS and PFS compared to TNM stage and treatment. Moreover, the prognostic model performed well across all time frames (1-, 3-, and 5-year) with regards to the IDI and NRI in comparison to the TNM stage, and clinical treatment.

**Conclusion:** The simple, convenient and low-cost prognostic model we developed demonstrated favorable performance for predicting OS and PFS in OSC patients, which may serve as a useful tool for physicians to provide personalized survival prediction for OSC patients.

## Introduction

Osteosarcoma (OSC) is the most common bone cancer, as well as one of the most common primary malignancies among children and adults^1^, ^2^. The incidence of OSC is 2-3/million/year in the general population, though epidemiologic statistics indicate that the incidence and mortality of OSC have been increasing at approximately 1.4% per year^3^. OSC mostly develops in the long bone near the epiphyseal growth plate of the extremities. The most common sites include the distal femur, proximal tibia, and proximal humerus. OSC is characterized by a high tendency to metastasize and local recurrence^4^. The incidence ratio of male to female is 1.4^5^.

Despite multidisciplinary therapies, including surgical excision, radiotherapy, and chemotherapy^6^, many OSC patients still experience tumor recurrence and metastasis, which results in poor prognosis and low survival rates among this group of patients^2^, ^7^. The presence or absence of metastasis, local recurrence, chemotherapy regimen, chemotherapy response, patient characteristics, tumor staging, tumor characteristics, and neoadjuvant tumor cell destruction percentage has an effect on prognosis^1^, ^7^. Specifically, metastasis has the greatest impact on prognosis. The overall survival rate of patients with metastatic disease is only about 20 - 30%, compared to 70 - 80% for non-metastatic patients^8^.

Clinical staging is a common method of assessing risk of OSC^9^. However, clinical staging systems for OSC, which include the American Joint Committee on Cancer (AJCC) staging and Enneking staging are only able to provide a rough assessment of the clinical risk for OSC based on pathological grade, tumor size, and metastasis. However, survival differs among patients with the same tumor stage^10^. These results indicate that the traditional staging system is inadequate for predicting the survival of cancer patients without considering other prognostic factors (such as clinical characteristics or blood biomarkers). Therefore, it is necessary to explore more reliable prognostic indicators to remedy shortcomings of the staging system, as well as to improve the prediction of clinical outcomes for patients with OSC.

Blood-based liquid biopsy has emerged as a useful tool for diagnosis and the prediction of outcome in patients with cancer. A variety of blood biomarkers have been studied with regards to the diagnosis and follow-up of OSC progression and recurrence, including alkaline phosphatase (ALP) and lactate dehydrogenase (LDH). Among the different blood biomarkers tested, ALP has been shown to have the most diagnostic value for OSC, and has been shown to be positively correlated with tumor volume, which has an additional useful prognostic significance^11^. Thus far, the clinical characteristics in combination with blood-based biomarkers were used to predict a prognosis of OSC had little been reported.

Thus, the retrospective study aimed to construct a prognostic model based on clinical characteristics and blood biomarkers for predicting the overall survival (OS) and progression-free survival (PFS) in patients with OSC, as well as to assess its incremental predictive ability in traditional TNM stage and clinical treatment of individual OS and PFS.

## Materials and methods

### Patient selection and data collection

Patients hospitalized and treated at the Sun Yat-sen University Cancer Center (SYSUCC) between January 2010 and December 2019 were consecutively enrolled into this present retrospective study. The inclusion criteria were as follows: (1) histologically confirmed OSC; (2) patients did not receive any anti-cancer treatment; (3) cancer-specific survival: alive or dead due to cancer. The exclusion criteria: (1) the existing malignancy other than OSC, alone or in combination with OSC; (2) Incomplete clinical information, laboratory data, and follow-up data. This study was granted approval by the Clinical Research Ethics Committee of the Sun Yat-sen University Cancer Center(B2022-481; Date of Approval: August 5, 2022). Since the study was retrospective in nature, the Institutional Review Board waived written informed consent. In addition, this study was carried out according to the principles of the Declaration of Helsinki.

Baseline clinical characteristics were collected from the patients' medical records, which included age, gender, smoking status, family history, tumor site, tumor size, tumor border, clinical treatment, and the eighth edition of the American Joint Committee on Cancer (AJCC) tumor-node-metastasis (TNM) staging^12^. The pretreatment blood-routine biomarkers were collected within one week before administering anti-cancer treatment. The biomarkers that were collected included white blood cell (WBC), neutrophil (N), lymphocyte (L), monocyte (M), platelet (PLT), neutrophil / lymphocyte ratio (NLR), lymphocyte / monocyte ratio (LMR), platelet/lymphocyte ratio (PLR), derived neutrophil-to-lymphocyte ratio (dNLR), prognostic nutritional index (PNI)^13^, red blood cell (RBC), hemoglobin (HGB), serum phosphorus (IP^3+^), serum calcium (Ca^2+^), serum magnesium (Mg^2+^), alanine aminotransferase (ALT), aspartate aminotransferase (AST), AST / ALT ratio (SLR), alkaline phosphatase (ALP), lactic dehydrogenase (LDH), glutamyl transpeptidase (GGT), total protein (TP), albumin (ALB), globulin (GLOB), C-reactive protein (CRP), ALB / CRP ratio (ACR), total bile acid (TBA), urea, creatinine (CRE), cystatin C (Cys-C), uric acid (UA), total cholesterol (CHO), triglycerides (TG), high density lipoprotein cholesterol (HDL-C), low density lipoprotein cholesterol (LDL-C), LDL-C / HDL-C ratio (LHR), apolipoprotein AI (APOA), apolipoprotein B (APOB), APOA / APOB ratio (ABR), glucose (GLU).

### Patients follow up

Data relevant to patient demographics and laboratory test data were abstracted from the electronic medical record. Follow-up was done by telephone or outpatient service, and the deadline for follow-up was April 2022. Overall survival (OS) was measured from the date of diagnosis until death due to cancer or the end point of the study. Progression-free survival (PFS) was calculated from date of the objective disease progression or death or the date of the last follow-up.

### Statistical analysis

Patients' characteristics were shown as frequencies (percentages) for categorical variables and mean ± standard deviation (SD) for continuous variables. We used the t-test or Wilcoxon test to compare mean. LASSO-Cox regression algorithm was adopted to select the most useful prognostic factors related to OS and constructed a novel prognostic model. Subsequently, Harrell's C-index (C-index), time-dependent ROC (td-ROC) curves^14^, decision curve analysis (DCA)^15^, net reclassification improvement index (NRI), and integrated discrimination improvement index (IDI)^16^ were used to compare the prognostic performances of the novel prognostic model with TNM stage and clinical treatment. DCA was utilized to evaluate clinical validity of the prognostic model and quantifying the net benefits at different threshold probabilities 17. The NRI assessed the ability of a new model to reclassify subjects into binary event or no-event categories compared to an older model. The IDI index quantified the improvement in average sensitivity without reducing the average specificity of a new model compared to the older model^18^. The correlation between the novel prognostic model, TNM stage, and clinical treatment was assessed using by Pearson's correlation coefficient. In addition, we constructed a nomogram that integrates the prognostic model risk score, TNM stage, and clinical treatment that may assist in individual survival prediction of OSC patients. Internal validation and calibration of the nomogram were performed via bootstrap resampling procedure. Finally, according to the risk score, OSC patients were classified into low-risk groups and high-risk groups, Kaplan-Meier method was used to compare the two groups in terms of PFS and OS. Statistical analyses were performed with R programing language (version 3.6.1) and Graph Pad Prism (version 5.0, San Diego, CA, USA), the results were considered statistically significant if the P value of less than 0.05.

## Results

### Demographic characteristics of the enrolled patients

Overall, 158 patients with osteosarcoma (OSC) were included in this retrospective study. 92 (58.2%) of these patients were male, and 66 (41.8%) were female. The median age was 16 years (95% confidence interval [CI], 15-18). According to 8th edition of the AJCC TNM stage criteria, the number of patients in stages I&II and III&IV was 121 (76.6%) and 37 (23.4%), respectively. The median OS was 32.5 months (95% CI, 30.4-36.2), The median PFS was 23.3 months (95% CI, 17.9-28.7). The baseline characteristics of the total OSC patients were shown in Table 1.

### The correlation between clinical characteristics and blood biomarkers

The correlation between clinical characteristics and blood biomarkers were shown in supplement table 1. The numbers in the table shown the Pearson's correlation coefficient between different variables. We found that different clinical characteristics were significantly associated with different blood biomarkers. Age was the most correlated with blood biomarkers, which significantly correlated with 20 (PLT, IP^3+^, ALT, SLR, ALP, GGT, Urea, CRE, UA, Cys-C, LDH, GLU, TG, CHO, HDL-C, LDL-C, LHR, APOA, APOB, and ABR) out of 39 blood biomarkers.

### Establishment of a prognosis model for OS and PFS

**Figure 1:**
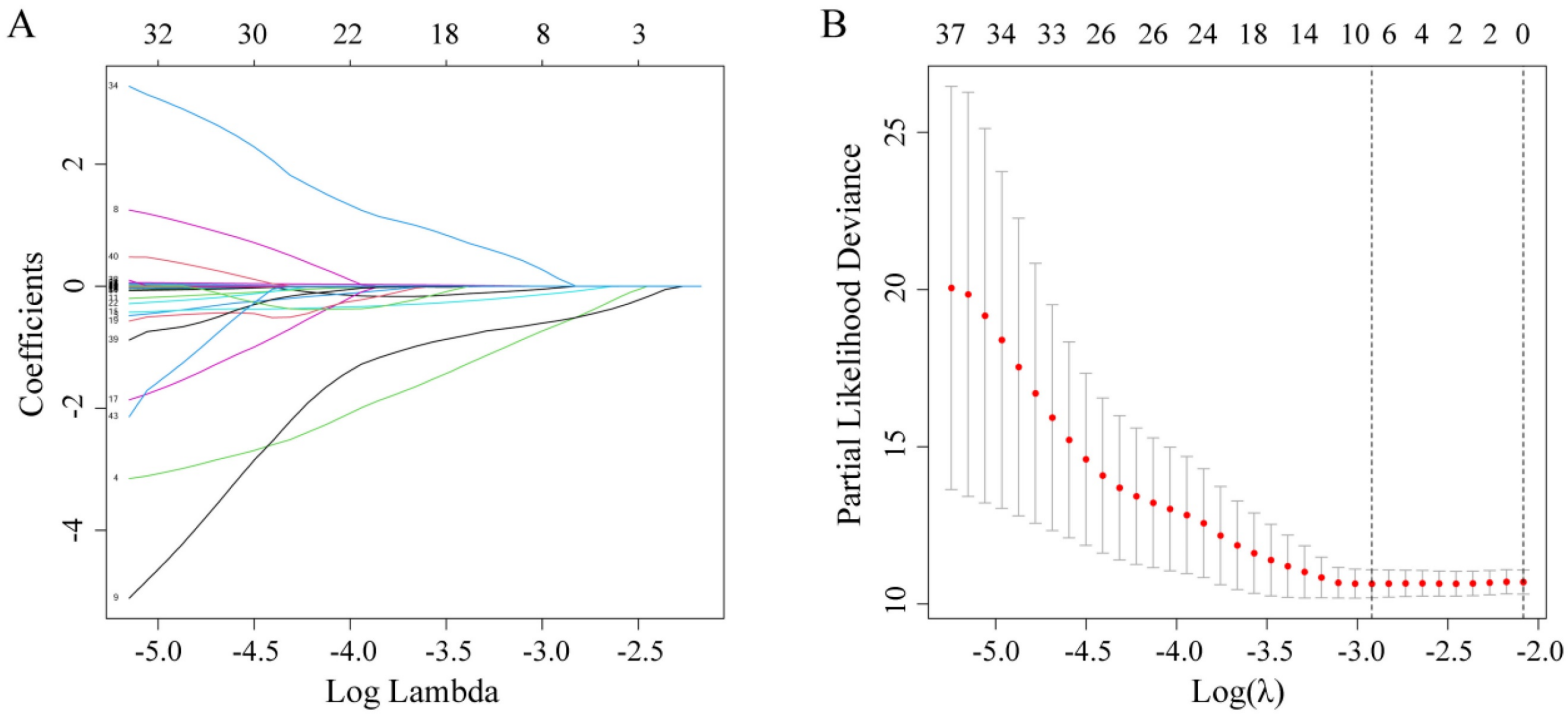
Potential predictors selection using LASSO-Cox regression analysis. (A) The changing trajectory of each variable in LASSO-Cox regression analysis; (B) Selection of the optimal lambda value with the minimum partial likelihood deviance.

Firstly, by using the LASSO-Cox regression analysis (Figure 1A), the optimal lambda value (lambda = 0.054) was obtained via minimum criteria (Figure 1B), and its corresponding six predictors (gender, family history of cancer, monocyte, RBC, Cys-C, and LDH) were correlated with OS. Finally, a simple prognostic model consisting of the six predictors were screened out in the LASSO-Cox regression analysis and were then generated risk score based on the regression coefficients. The prognostic model risk score was calculated using the following formula: risk score = -(0.0247×gender) - (0.6294×family history of cancer) - (0.5652×monocyte) - (0.1119×RBC) + (0.0019 ×LDH) + (0.1293×Cys-C). In this formula, the following dichotomous variables: gender (Male=1, Female=0), family history of cancer (yes=1, no=0), the other continuous variables value represents their respective serum levels. This formula was applied to calculate each patient's risk score.

### The comparison between the prognostic model with TNM stage and clinical treatment

**Figure 2:**
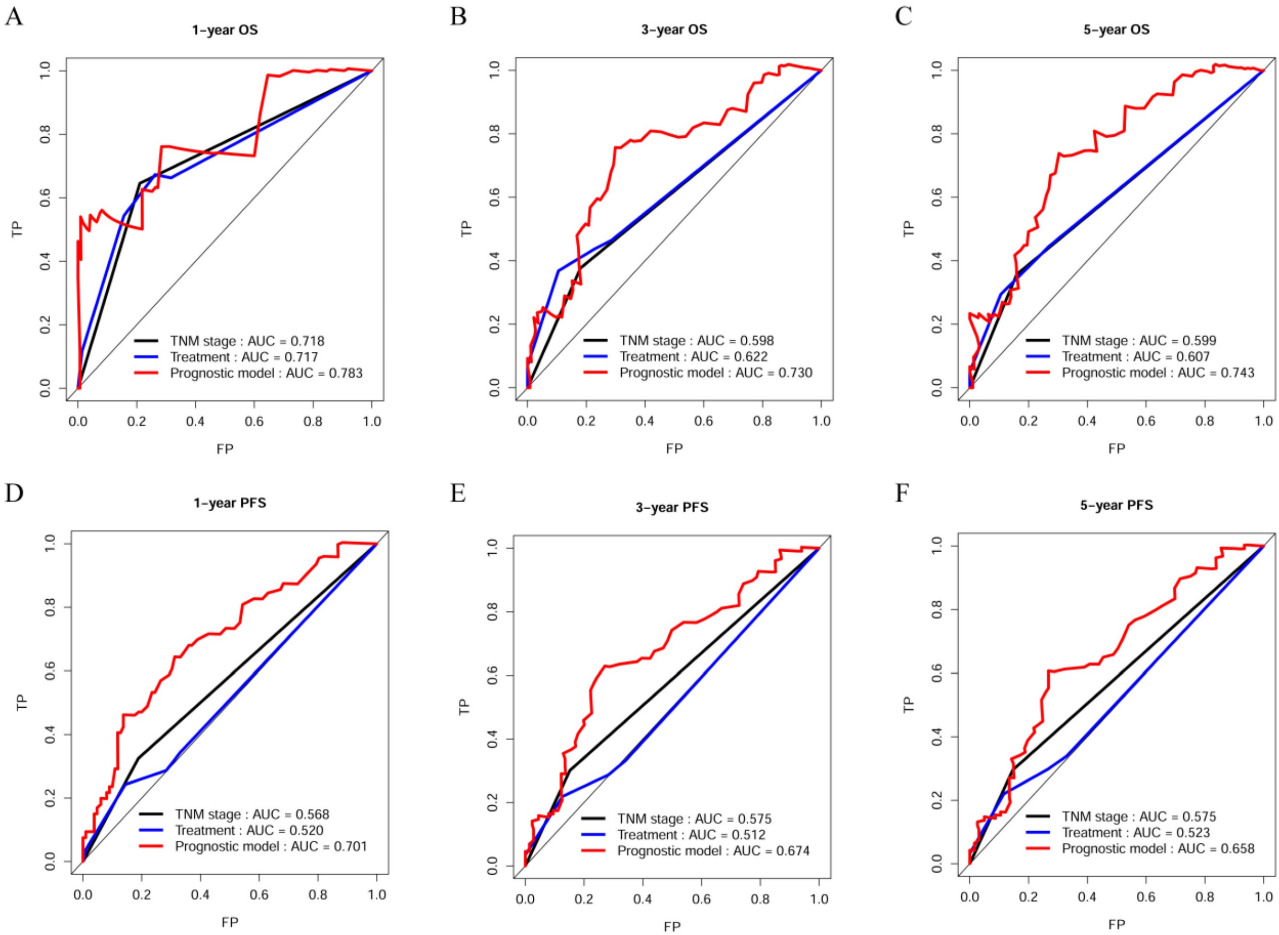
Time dependent ROC curves analysis of the novel prognostic model, TNM stage, and clinical treatment in OS and PFS. (A-C) Time-dependent AUC curves of the prognostic model, TNM stage, and clinical treatment at 1-year OS, 3-year OS, and 5-year OS; (D-F) Time-dependent AUC curves of the prognostic model, TNM stage, and clinical treatment at 1-year PFS, 3-year PFS, and 5-year PFS.

**Figure 3:**
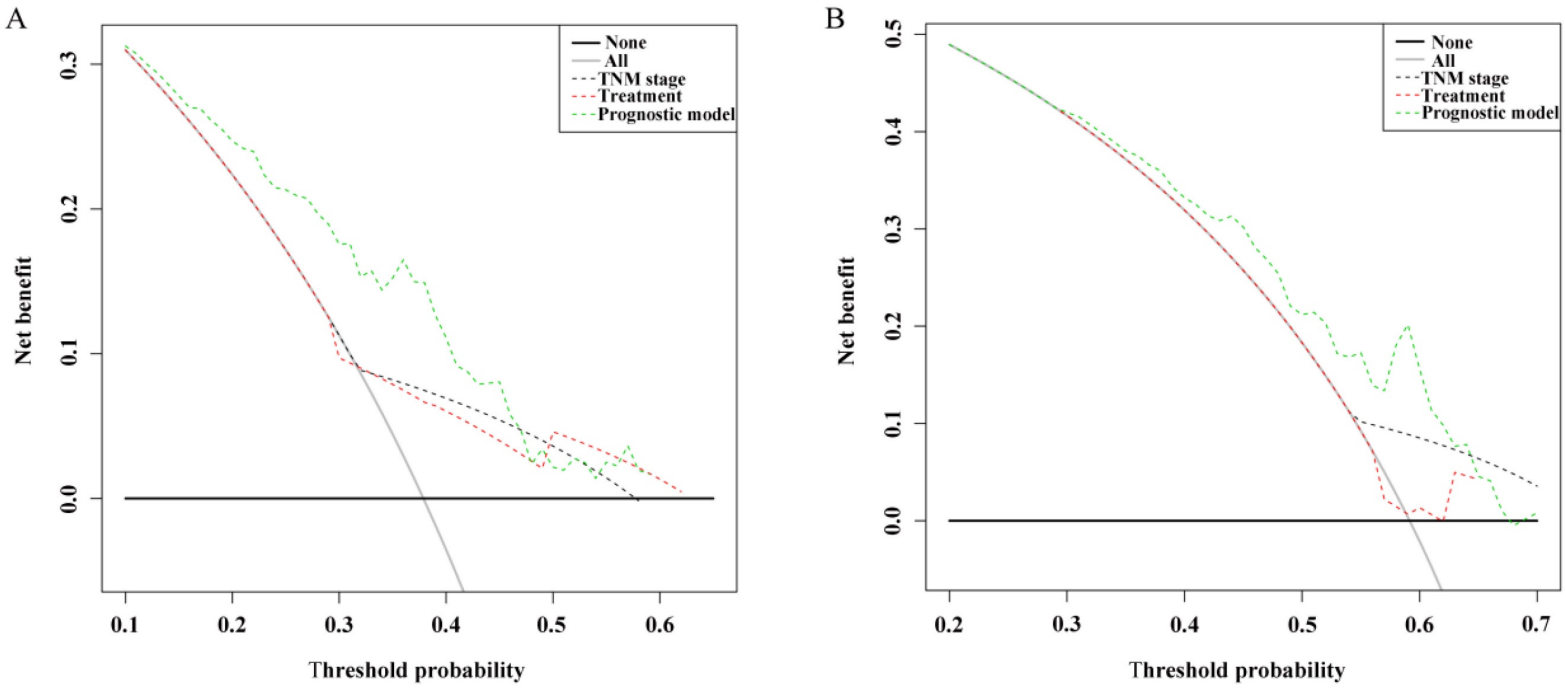
Decision curve analysis of the novel prognostic model, TNM stage, and clinical treatment in OS and PFS. (A) Decision curve analysis of OS; (B) Decision curve analysis of PFS.

The C-index, td-ROC curve, DCA, NRI, and IDI were used to compare the prognostic performances of the novel prognostic model with TNM stage and clinical treatment. Firstly, the C-index was calculated and compared to the C-index of the three predictive signatures (Table 2). For OS, the C-index of the prognostic model was 0.713 (95% CI = 0.630-0.795), which was significantly higher than that in TNM stage [0.590 (95% CI = 0.518-0.663), *P* = 0.011] and clinical treatment [0.604 (95% CI = 0.521-0.687), *P* = 0.020]. With regards to PFS, the C-index of the novel prognostic model was 0.636 (95% CI = 0.577-0.696), which was shown to be significantly higher compared to that of the TNM stage [0.552 (95% CI = 0.503-0.600), *P* = 0.022] and clinical treatment [0.517 (95% CI = 0.461-0.574), *P* = 0.001]. Secondly, the td-ROC curves were plotted, and the area under ROC curves (AUCs) of the three predictive signatures were calculated. For OS, the AUCs of 1-, 3-, and 5-year were 0.783, 0.730, and 0.743, respectively. And for PFS, the AUCs of 1-, 3-, and 5-year were 0.701, 0.674, and 0.658, respectively. The AUCs of the novel prognostic model were higher compared to that of TNM stage and clinical treatment, with regards to both OS and PFS at 1-, 3-, and 5-year. (Figure 2). Thirdly, the DCA demonstrated that within the most reasonable threshold probability range of OS and PFS, the novel prognostic model had a higher overall net benefit than the TNM stage and clinical treatment (Figure 3). Finally, both NRI and IDI calculations were obtained at 1-, 3-, and 5-year, and were utilized to compare alternative prognostic indices of our prognostic model with TNM stage and clinical treatment. Positive value represents better accuracy while negative value represents worse accuracy. The results were presented in Table 3. For OS, NRI analysis indicated that the prognostic model had higher predictive power compared to that of the TNM stage and clinical treatment both at 1-, 3-, and 5-year OS survival. IDI analysis also indicated that that the discrimination ability of the novel prognostic model was higher than that of the TNM stage and clinical treatment. In addition, similar results also indicated that the novel prognostic model had improved performance in predicting PFS for OSC patients than others.

### Establishment of predictive nomogram

**Figure 4:**
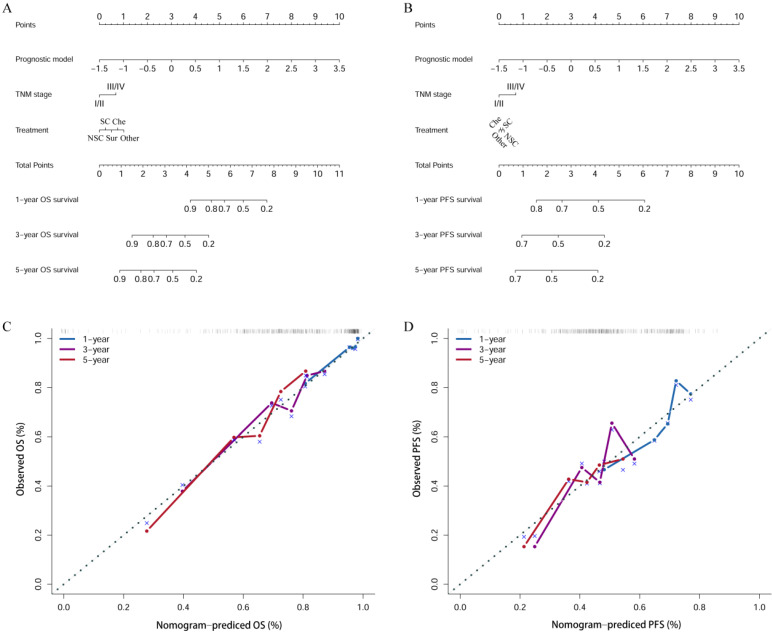
The nomogram plots for estimating OS (A) and PFS (B) at 1, 3, and 5 years. Calibration curves of the nomogram for OS (C) and PFS (D). NSC: neoadjuvant therapy + surgery + chemotherapy; SC: surgery + chemotherapy; Che: chemotherapy.

The nomogram incorporated the prognostic model risk score, TNM stage, and clinical treatment to quantitative analysis the 1-, 3-, and 5- years OS (Figure 4A) and PFS (Figure 4B) survival probability for each OSC patient. The points of the factors indicate their corresponding contribution to survival probability. And the calibration curves indicated the nomogram-predicted 1-, 3-, 5-year OS (Figure 4C) and PFS (Figure 4D) were matched well with actual 1-, 3-, 5-year OS and PFS.

### Performance of the prognostic model in stratifying risk

**Figure 5:**
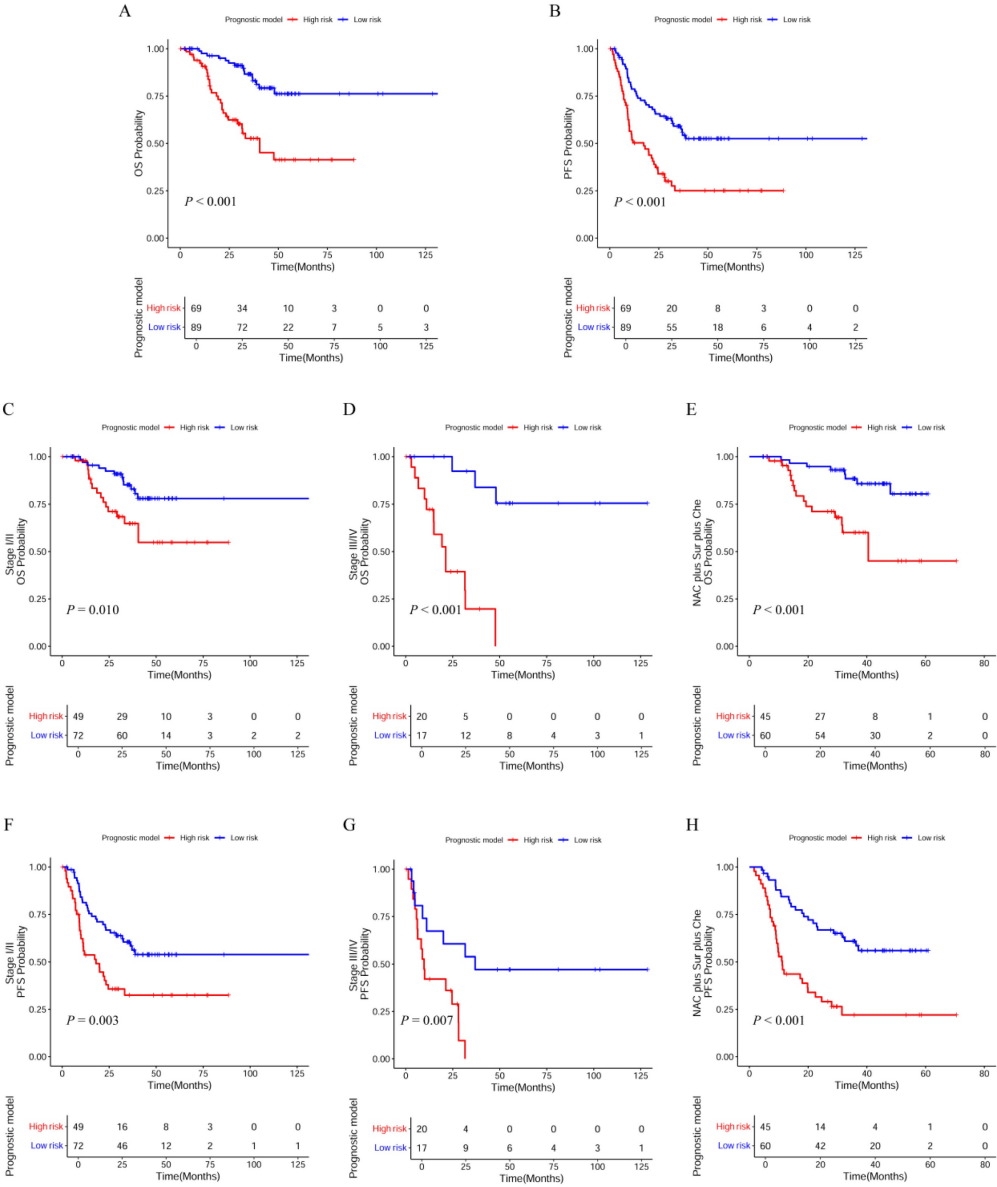
Kaplan-Meier (K-M) curves with log-rank p-values of the prognostic model. (A, B) K-M curves for OS and PFS of low-risk and high-risk patients basing on the prognostic model; (C, D) K-M curves of OS prognostic value for the prognostic model in the full groups with tumor stage; (E) K-M curves of OS prognostic value for the prognostic model in the full groups with clinical treatment; (F, G) K-M curves of PFS prognostic value for the prognostic model in the full groups with tumor stage; (H) K-M curves of PFS prognostic value for the prognostic model in the full groups with clinical treatment. NAC: neoadjuvant therapy; Sur: surgery; Che: chemotherapy.

According to the risk scores, the patients were classified into low-risk groups and high-risk groups, differences in survival between the two groups were tested using the Kaplan-Meier method and compared by the log rank tests. Patients in the high-risk group (risk score ≥ -0.27) tended to have a worse OS (Figure 5A; *P* < 0.001) and PFS (Figure 5B; *P* < 0.001) than those in the low-risk group (risk score < -0.27). Additionally, we wanted to test whether the prognostic model would be able to make up for the current deficiencies of the AJCC TNM stage and clinical treatment. Next, patients were factitiously stratified into the early stage (stage I/II), the late stage (stage III/IV), and received the same clinical treatment (neoadjuvant therapy plus surgical resection plus chemotherapy). Kaplan-Meier curve indicated that high-risk patients in the early stage had significantly lower OS (Figure 5C;* P* = 0.010) and PFS (Figure 5F; *P* = 0.003) compared to low-risk patients. In the late stage, the OS (Figure 5D; *P* < 0.001) and PFS (Figure 5G; *P* = 0.007) in the low-risk and high-risk groups also displayed significant difference. In the patients received the same clinical treatment, the result suggested a poorer prognosis in the high-risk group both in OS (Figure 5E; *P* < 0.001) and PFS (Figure 5H; *P* < 0.001).

### Differences between the high-risk and low-risk group in the 6 selected predictors

**Figure 6:**
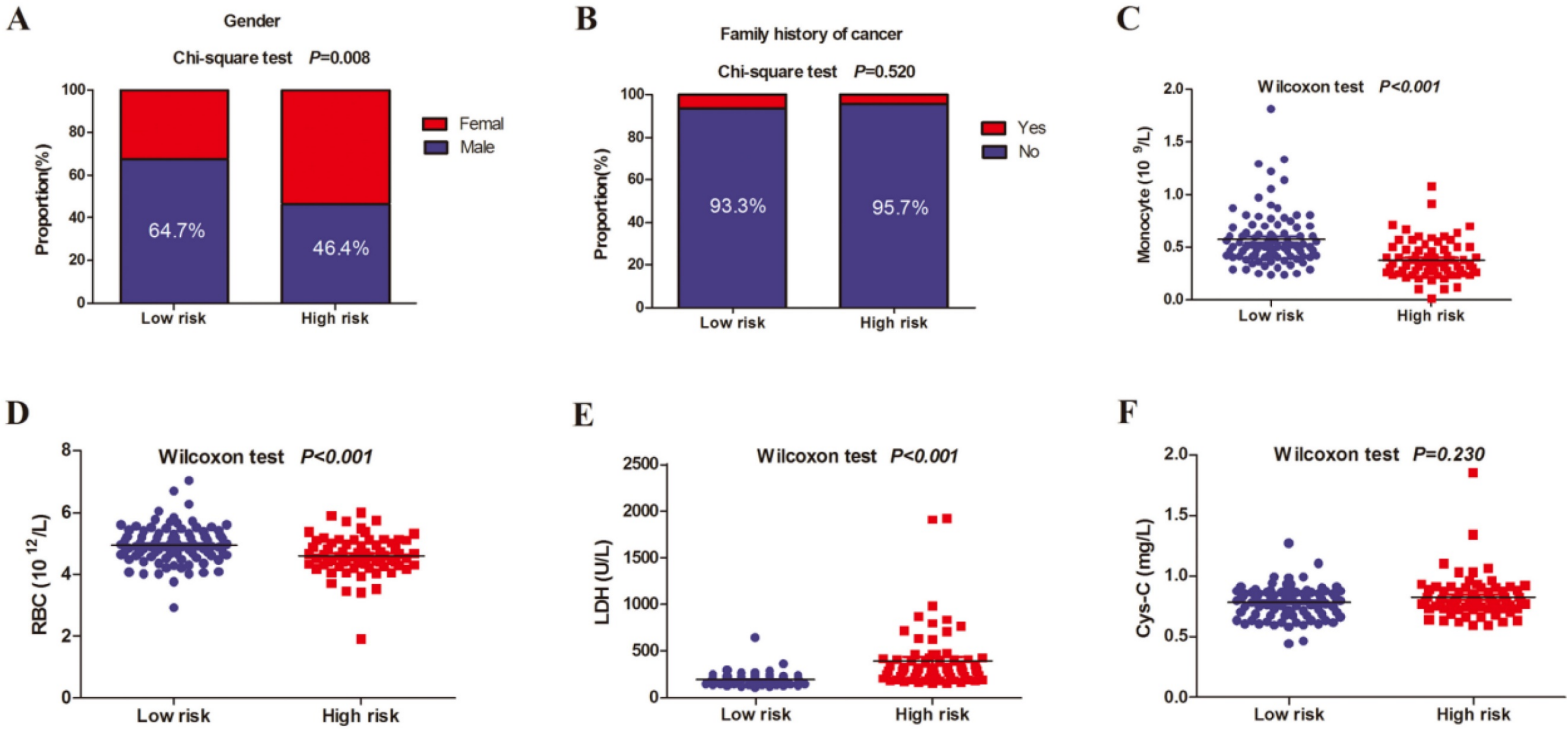
Differences between the high-risk and low-risk group in the gender, family history of cancer, monocyte, RBC, LDH, and Cys-C. (A) gender; (B) family history of cancer; (C) monocyte; (D) RBC; (E) LDH; (F) Cys-C.

Figure 6 showed the composition of the low-risk and high-risk patients by gender and family history of cancer, and compared serum values of monocyte, RBC, LDH, and Cys-C between the two groups. There was significant statistical difference in gender, monocyte, RBC, and LDH between low-risk and high-risk patients. Monocyte and RBC levels in low-risk were higher than the high-risk group.

### Correlation analysis between the prognostic model, TNM stage, and clinical treatment

**Figure 7:**
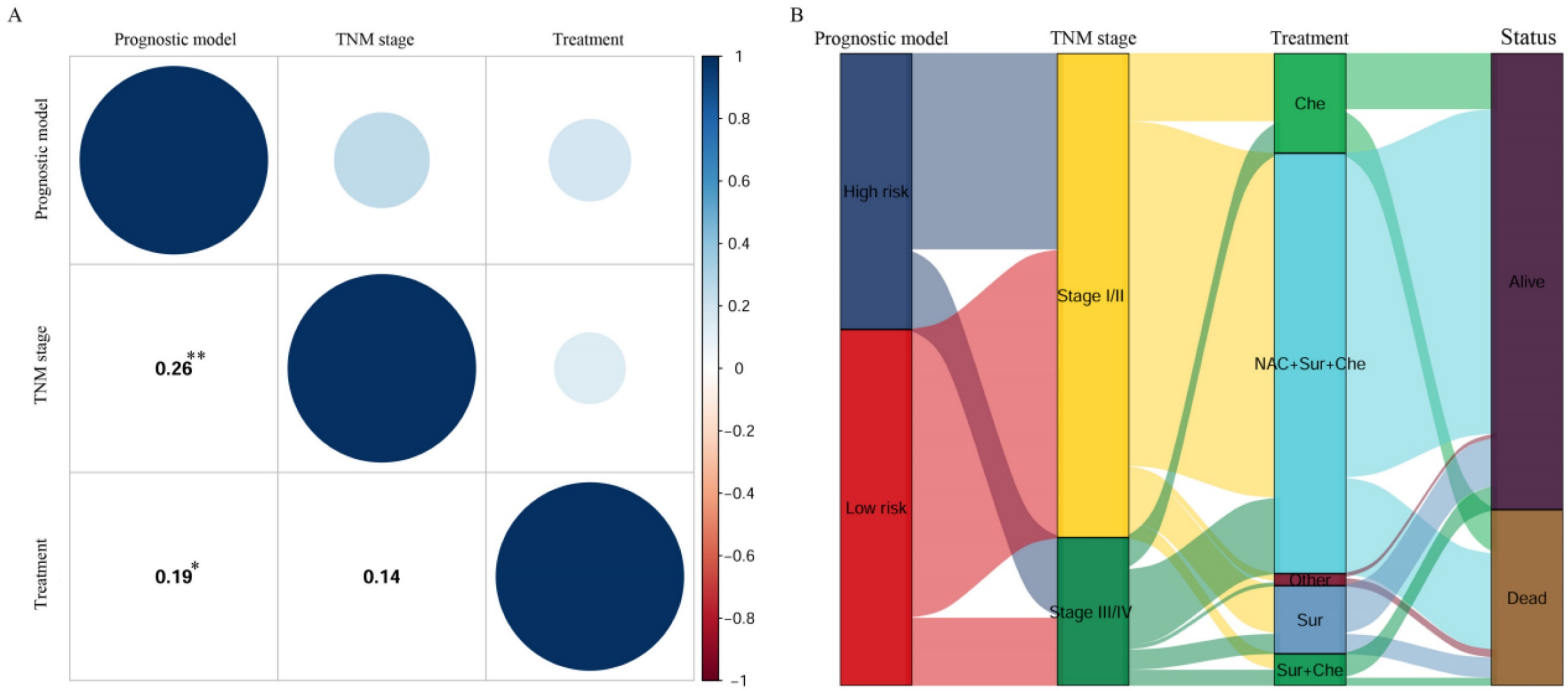
(A) The correlations between the prognostic model, TNM stage, and clinical treatment; (B) The sankey plot showed the patients' transfers between the prognostic model, TNM stage, treatment and survival status. NAC: neoadjuvant therapy; Sur: surgery; Rad: radiotherapy; Che: chemotherapy.*:* P* < 0.05; **:* P* < 0.01

To investigate the relationship between the prognostic model, TNM stage, and clinical treatment, a Pearson correlation coefficient (PCC) analysis was performed (Figure 7A), where the red represented negative correlation and the blue represented positive correlation, and the circle size represented the size of correlation coefficient. The results indicated that the prognostic model was significantly and positively correlated with TNM stage (PCC = 0.26, *P* = 0.001), as well as clinical treatment (PCC = 0.19, *P* = 0.018). In addition, prognostic model, TNM stage, clinical treatment, and status were shown in the Sankey plot and there was a positive regulatory relationship between prognostic model, TNM stage, clinical treatment, and status (Figure 7B).

## Discussion

In this research, we focused on the relationship between the clinical characteristics and blood biomarkers and prognostic value for patients with OSC. The LASSO-Cox regression method was applied to build a model to predict OS and PFS in OSC patients. Our prognostic model was used to predict OS and PFS in OSC patients with a high accuracy and stability, which outperformed the traditional TNM stage and clinical treatment.

Based on results from the LASSO-Cox regression, six prognostic factors (gender, family history of cancer, monocyte, RBC, LDH, and Cys-C) were screened out and then incorporated into the novel predictive model for OSC patients. It had been reported that gender, monocyte, and LDH were related to prognosis of OSC, while levels of RBC and Cys-C were known to be related to the prognosis of other malignant tumors. Monocyte played significant roles in the establishment of the immune microenvironment of OSC. The higher monocyte levels could inhibit the metastasis of OSC, and had longer overall survival times^19^, ^20^. The serum level of LDH was known to reflect systemic cancer burden, growth, survival and invasive potential^21^. Previous studies had shown that serum LDH level was associated with the prognosis of OSC patients^22^, ^23^. Family history of cancer was associated with developing cancer, furthermore, it was recognized as an important prognostic factor in several cancers^24^-^27^. RBC level was one of the RBC parameters, and a reduced preoperative RBC count may reflect poor liver function, which could affect survival of patient survival^28^, ^29^. Lu* et al.* reported that in patients with primary liver cancer, patients whose preoperative RBC counts were lower than normal demonstrated a lower OS rate compared to patients whose preoperative RBC counts did not decrease^30^. Cys-C was an endogenous marker of glomerular filtration rate, numerous studies had shown that abnormal serum Cys-C levels can be used as a prognostic and diagnostic indicator for several malignancies^31^-^34^. All of the data indicated that these prognostic factors were closely related to tumor development, and suggested that our analysis had reliable prognostic value.

In order to evaluate whether our model was able to remedy the deficiencies of the TNM stage and clinical treatment in the prognostic assessment of OSC patients, the OSC patients were divided into the low-risk and high-risk groups based on their prognostic model risk scores. Kaplan-Meier survival curves showed that the high-risk groups had lower OS and PFS in OSC patients with stage I/II, stage III/IV, and clinical treatment. Thus, the results reminded us that even for patients at the same stage, and received the same clinical treatment, high-risk patients will likely require more intense treatment. Moreover, the results also indicated that our model could remedy deficiencies of the TNM stage, and enhance the predictive power of the TNM stage. Improved prediction of individual prognosis could aid clinicians in many ways, including consulting patients, choosing personalized treatment, and arrange patient follow-up.

This study had several advantages compared to previous studies^35^-^37^: Firstly, in the past, most prognostic factors were single indicators, but our study had included more potential prognostic factors than the previous studies. And this prognostic model was established based on clinical characteristics and clinical laboratory blood tests available in most clinical settings. So, it is simple, convenient and low-cost for clinical application, especially the application of grass-roots hospitals. Second, we utilized the new algorithm LASSO-Cox regression analysis to develop a prognostic model as a statistical method to filter variables and establish a prognostic model. This allowed us to adjust the model's overfitting to avoid extreme predictions, thereby significantly improving the prediction accuracy. This method has been applied across many studies^38^-^40^. Thirdly, in this study, we utilized multiple methods to compare the predictive accuracy and discriminative ability of the novel prognostic model with TNM stage and clinical treatment. In addition, these results all demonstrated that our model outperformed compared to others. Lastly, the endpoint of this study was OS and PFS, so this model could achieve better clinical application.

The research still presented some limitations: 1. Selection bias was unavoidable due to the retrospective design of this research. So, its calculated predictive value was for clinicians' reference only. 2. The sample size was relatively small, single-centre data collection, lack of external validation which limit generalizability of the prognostic model. 3. The model was non-specific prediction tool for OSC patients, and may lack certain specificity. Some specific biomarkers may be incorporated into the prognostic model to improve the specificity, such as immunohistochemical markers^41^, radiomics^42^, ^43^, and the recently newly applied non-coding RNAs^44^, ^45^. 4. The subtype of OSC was reported as a prognostic marker, different subtypes of OSC had different survival outcomes^46^. As this study failed to obtain OSC subtypes for each patient, in was not included in our study as a potential prognostic marker. 5. We collected data only for the initial diagnosis and did not dynamically monitor the entire course of the patient. Thus, we could not know the significance of biomarkers for prognosis of the patient after each treatment. 6. We did not compare our model with previously developed and validated prognostic models. This study was a retrospective study, we cannot obtain the data contained in these models from our electronic medical records. Therefore, we did not compare our prognostic model with previously developed prognostic models. The AJCC TNM staging system of osteosarcoma is the most widely used method for survival prediction. So, we replaced the previously developed models with AJCC TNM staging system and compare it with our model. Despite these shortcomings, the prognostic model was effective and could help predict prognosis of OSC patients, thus providing clinicians with a more practical and convenient tool for individualized treatment decision making and survival assessment at the initial diagnosis.

## Conclusions

In conclusion, we successfully constructed a 6 clinical characteristics and blood biomarkers-based prognostic model for OSC patients. This model outperformed TNM stage and clinical treatment in predicting OS and PFS in OSC patients. Low-cost and satisfactory stability of this prognostic model may act as a useful tool for physicians to provide consultation, personalized survival prediction for OSC patients. But, the properties of our prognostic model required verification before wide variety of clinical applications.

## Supplementary Material

Supplementary table.

## Figures and Tables

**Table 1:** Demographics and clinical characteristics of OSC patients

Variable	No. (%) or mean ±standard deviation (SD)
Characteristics	
Age (years)	22.1 ± 15.4
Gender	
Male	92 (58.2%)
Female	66 (41.8%)
Smoke	
Yes	7 (4.4%)
No	151 (95.6%)
Family history of cancer	
Yes	9 (5.7%)
No	149 (94.3%)
Tumor site	
Skull	19 (12.0%)
Trunk	8 (5.1%)
Extremities	131 (82.9)
Treatment	
NAC plus Sur plus Che	105 (66.5%)
Sur plus Che	8 (5.1%)
Sur	17 (10.8%)
Che	25 (15.8%)
Other	3 (1.8%)
TNM stage^a^	
I&II	121 (76.6%)
III&IV	37 (23.4%)
	
Laboratory data	
WBC (10^9^/L)	8.18 ± 3.19
Neutrophil (10^9^/L)	5.53 ± 2.96
Lymphocyte (10^9^/L)	1.99 ± 0.66
Monocyte (10^9^/L)	0.49 ± 0.25
PLT (10^9^/L)	310.45 ± 83.19
NLR	3.11 ± 2.48
LMR	5.46 ± 8.13
PLR	171.34 ± 72.56
dNLR	2.29 ± 2.05
PNI	54.69 ± 6.46
RBC (10^12^/L)	4.79 ± 0.65
HGB (g/L)	131.20 ± 19.61
IP^3+^ (mmol/L)	1.39 ± 0.23
Ca^2+^ (mmol/L)	2.40 ± 0.68
Mg^2+^ (mmol/L)	0.90 ± 0.08
ALT (U/L)	16.98 ± 12.57
AST (U/L)	20.93 ± 8.34
SLR	1.70 ± 1.14
ALP (U/L)	470.73 ± 1038.01
LDH (U/L)	277.24 ± 241.36
GGT (U/L)	23.20 ± 16.33
TP (g/L)	74.31 ± 6.15
ALB (g/L)	44.71 ± 5.43
CRP (mg/L)	10.64 ± 20.24
ACR	52.34 ± 112.58
TBA (umol/L)	4.98 ± 5.67
Urea (mmol/L)	4.40 ± 1.51
CRE (umol/L)	52.83 ± 17.91
Cys-C (mg/L)	0.80 ± 0.16
UA (umol/L)	338.15 ± 93.32
CHO (mmol/L)	4.06 ± 1.01
TG (mmol/L)	1.19 ± 0.65
HDL-C (mmol/L)	1.19 ± 0.30
LDL-C (mmol/L)	2.46 ± 0.89
LHR	2.19 ± 0.95
APOA (g/L)	1.21 ± 0.23
APOB (g/L)	0.80 ± 0.23
ABR	1.63 ± 0.53
GLU (mmol/L)	5.17 ± 1.00

a: TNM stage was classified according to the AJCC 8th TNM staging system; Abbreviations: TNM: Tumor Node Metastasis; NAC: neoadjuvant therapy; Sur: surgery; Rad: radiotherapy; Che: chemotherapy; PLT: platelet; NLR: neutrophil / lymphocyte ratio; LMR: lymphocyte / monocyte ratio; PLR: platelet / lymphocyte ratio; dNLR: derived neutrophil-to-lymphocyte ratio; PNI: prognostic nutritional index; RBC: red blood cell; HGB: hemoglobin; IP^3+^: serum phosphorus; Ca^2+^: serum calcium; Mg^2+^: serum magnesium; ALT: alanine aminotransferase; AST: aspartate aminotransferase; SLR: AST / ALT ratio; ALP: alkaline phosphatase; LDH: lactic dehydrogenase; GGT: glutamyl transpeptidase; TP: total protein; ALB: albumin; CRP: C-reactive protein, ACR: ALB / CRP ratio; TBA: total bile acid; CRE: creatinine; Cys-C: cystatin C; UA: uric acid; CHO: total cholesterol; TG: triglycerides; HDL-C: high density lipoprotein cholesterol; LDL-C: low density lipoprotein cholesterol; LHR: LDL-C / HDL-C ratio; APOA: apolipoprotein A1; APOB: apolipoprotein B; ABR: APOA / APOB ratio; GLU: glucose.

**Table 2:** The C-index of OS and PFS for prognostic model, TNM stage, and treatment.

Survival prediction	C-index	95 CI%	*P*
For OS			
Prognostic model	0.713	0.630 - 0.795	
TNM stage	0.590	0.518 - 0.663	
Treatment	0.604	0.521 - 0.687	
Prognostic model vs TNM stage			0.011
Prognostic model vs Treatment			0.020
			
For PFS			
Prognostic model	0.636	0.577 - 0.696	
TNM stage	0.552	0.503 - 0.600	
Treatment	0.517	0.461 - 0.574	
Prognostic model vs TNM stage			0.022
Prognostic model vs Treatment			0.001

C-index = concordance index; *P* values are calculated based on normal approximation using function rcorrp.cens in Hmisc package.

**Table 3:** A comparison of discriminatory ability of prognostic model with TNM stage and treatment using NRI and IDI for OS and PFS.

	1-Year				3-Year				5-Year			
	NRI	*P*	IDI%	*P*	NRI	*P*	IDI	*P*	NRI	*P*	IDI	*P*
For OS												
Prognostic model vs TNM stage	0.378	0.149	0.200	<0.01	0.202	0.060	0.100	0.030	0.129	0.308	0.077	0.308
Prognostic model vs Treatment	0.344	0.239	0.194	0.040	0.151	0.239	0.081	0.209	0.418	0.01	0.131	0.020
												
For PFS												
Prognostic model vs TNM stage	0.193	0.100	0.058	0.060	0.161	0.209	0.054	0.109	0.063	0.557	0.042	0.318
Prognostic model vs Treatment	0.345	0.060	0.076	<0.01	0.253	0.090	0.075	0.010	0.280	0.269	0.054	0.209
