# Supplementary material for: A novel prognostic model to predict prognosis of patients with osteosarcoma based on clinical characteristics and blood biomarkers
Source: J Cancer. 2025 Mar 10;16(7):2075–86. doi: 10.7150/jca.105590 (PMC12036093; doi:10.7150/jca.105590)
Supplement: Supplementary file 1 — Supplementary table. [file jcav16p2075s1.pdf]

Supplement table 1. The correlation between clinical characteristics and blood biomarkers

	Age	Gender	Smoke	Family history of cancer	Tumor site	Treatment	TNM stage
WBC	0.00	0.17*	-0.07	0.03	-0.01	-0.18*	-0.04
Neutrophil	0.01	0.15*	-0.05	0.02	0.02	-0.18*	-0.02
Lymphocyte	-0.07	-0.01	-0.11	0.02	-0.11	-0.03	-0.10
Monocyte	0.05	0.26**	-0.07	0.09	-0.11	-0.126	-0.09
PLT	-0.25**	-0.09	-0.08	0.20*	0.15*	-0.11	0.03
LMR	-0.04	-0.14*	-0.03	-0.05	0.07	0.00	-0.03
NLR	0.03	0.09	0.00	0.00	0.08	-0.13*	0.00
dNLR	0.03	0.02	0.00	-0.01	0.07	-0.12	0.00
PLR	-0.11	-0.10	0.01	0.13	0.16*	-0.04	0.08
RBC	-0.06	0.24**	0.08	-0.19*	0.08	-0.19*	-0.12
HGB	0.11	0.37***	0.19*	-0.17*	-0.06	-0.21**	-0.13*
IP^{3+}	-0.60***	0.00	-0.24**	-0.10	0.26**	-0.12	-0.03
Ca^{2+}	-0.02	-0.09	-0.02	-0.06	-0.14*	-0.01	-0.05
Mg^{2+}	0.12	0.04	0.08	0.09	-0.09	0.01	-0.10
ALT	0.24**	0.19*	0.12	-0.10	-0.18*	-0.06	0.00
AST	-0.16*	-0.04	-0.05	-0.08	0.09	0.08	0.16*
SLR	-0.42***	-0.22**	-0.16*	0.08	0.22**	0.05	0.08
TBA	-0.03	0.05	0.02	0.07	0.03	-0.14*	-0.14*
ALP	-0.23**	0.05	-0.07	-0.04	0.14*	0.16*	0.17*
GGT	0.40***	0.27***	0.10	-0.10	-0.06	0.02	0.01
TP	-0.10	0.07	-0.06	-0.17*	0.28***	-0.23**	-0.20*
ALB	0.01	-0.02	-0.06	-0.22**	0.10	-0.08	-0.13
PNI	-0.03	-0.02	-0.11	-0.17*	0.03	-0.08	-0.16*
CRP	-0.09	0.14*	-0.07	0.16*	0.09	-0.10	0.01
ACR	-0.04	-0.12	-0.05	-0.11	-0.08	0.06	-0.10
Urea	0.33***	0.17*	0.11	-0.17*	-0.12	0.12	0.09
CRE	0.53***	0.37***	0.14*	-0.09	-0.24**	-0.04	-0.01
UA	0.15*	0.30***	0.11	-0.08	0.10	0.02	0.06
Cys-C	0.32***	0.22**	0.13*	-0.10	-0.04	0.12	0.02
LDH	-0.24**	-0.02	-0.09	0.13	0.20*	0.15*	0.25**
GLU	0.18*	0.03	0.03	0.09	0.03	0.06	0.01
TG	0.37***	0.09	0.21**	-0.09	-0.06	0.08	0.11
CHO	0.43***	-0.12	0.10	-0.09	-0.22**	0.19	0.03
HDL-C	0.14*	-0.18*	-0.10	-0.13*	-0.10	0.10	-0.04
LDL-C	0.36***	-0.11	0.06	-0.05	-0.17*	0.19*	0.04
LHR	0.24**	0.03	0.11	0.05	-0.11	0.14*	0.06
APOA	0.25***	-0.13*	-0.06	-0.13*	-0.10	0.08	0.01
APOB	0.40***	-0.03	0.14*	0.00	-0.18*	0.17*	0.05
ABR	-0.19*	-0.05	-0.16*	-0.10	0.06	-0.08	-0.04
